# Improved Understanding of Sugar Transport in Various Plants

**DOI:** 10.3390/ijms231810260

**Published:** 2022-09-06

**Authors:** Li-Qing Chen

**Affiliations:** Department of Plant Biology, University of Illinois Urbana-Champaign, Urbana, IL 61801, USA; lqchen77@illinois.edu

A few recent reviews have addressed progress and perspectives in the field of sugar transport in plants rather comprehensively [1,2,3,4,5], so this editorial will be confined to emphasizing the achievements of the publications in this Special Issue. This Special Issue is designed to enhance our understanding of sugar transport in plants. Sugar transport occurs within the cells and among the cells to communicate sugar across the plasma membrane or different subcellular compartments. Different types of sugar transporters play a vital role in mediating sugar transport. However, our great understanding of the function and regulation of sugar transporters is mainly based on findings from experiments on the model plant, *Arabidopsis thaliana*, although many discoveries about sugar transport in crops, such as maize and rice, have also been reported. More research about sugar transport from different plant species is needed to gain a better understanding of this process. Especially little is known about the phloem loading and unloading mechanisms that are used for most plants [2], and little is known about the function of sugar transport in many plants under different stress conditions.

In this Special Issue, we collected reports/reviews from research on lilies, sugarcane, grapes, and litchi fruit. These species have either a source-sink switch mechanism, different sugar storage organs or different sugar storage forms. Zeng et al. reported a correlation between the expression of sugar transporters and sugar content in the lily, a perennial bulbous plant in which underground organs and aboveground organs switch roles as source versus sink tissues depending on the stage of plant growth and development [6]. This switch is accompanied by spatial and temporal changes in the expression of sugar transporters, which is itself regulated by low temperatures, salt stress, and drought stress [6]. This study provides a deeper understanding of gene expression during this switch, although it remains elusive which and how individual genes are physiologically involved in this process. As pathogens rely on carbon source supply from hosts, sugar transporters have been reported to play a critical role in the process of feeding pathogens [1,7]. Akbar et al. compared the expression of genes associated with sugar metabolism, sugar transport, and sugar storage, and compared related protein accumulation between two sugarcane genotypes infected with the *Sugarcane mosaic virus* (SCMV), one type susceptible to SCMV (Badila) and the other resistant to SCMV (B-48) [8]. They found that fewer those genes and proteins were regulated in the SCMV-resistant cultivar compared to the SCMV-susceptible cultivar [8]. Specifically, glycosyl hydrolase 17, which is involved in plant immune responses, was highly upregulated in transcript and protein levels in the B-48 sugarcane, suggesting which might contribute to B-48’s SCMV resistance [8]. Although more research is needed to elucidate the molecular function of individual regulated genes, including sugar transporters, in SCMV resistance, this study provides insight into potentially important genes involved in SCMV resistance. We also have a limited understanding of the regulation of sugar transporters at the post-translational level. Cai et al. reported that hetero-dimerization (or hetero-oligomerization) of grape sucrose transporters VvSUC11 and VvSUC12 altered transport kinetics relative to homo-dimerization (or homo-oligomerization) of individual transporters [9]. In addition, they found that heteromerization of either VvSUC11 or VvSUC12 with VvSUC27 resulted in reduced transport activity of VvSUC27 [9]. Moreover, these three transporters were found to be colocalized to the central carpellary bundle in grape berries. However, *VvSUC11* and *VvSUC12* showed similar expression patterns positively correlated with developmental sugar accumulation, distinct from the expression pattern of *VvSUC27*, which was negatively correlated with developmental sugar accumulation of different tissues [9]. These data suggest regulation of sugar accumulation in grapes is achieved at different development stages of different tissues at least partially by regulating *VvSUC* expression and, in turn, adjusting the relative amount of homomers and heteromers with different activities in transporting sucrose. Further in vivo studies are required to test this mechanism.

In this Special Issue, there are two review articles. Hu et al. provided an overview of rice sugar transporter *SUT* and *SWEET* gene expression [10]. They summarized the physiological functions of rice SUT and SWEET transporters reported so far, from developmental functions to gene expression regulated by abiotic and biotic stresses [10]. This offers a starting point from which to understand rice SUC and SWEET transporters. Another paper submitted by Fan et al. reviewed sugar transport and metabolism and their involvement in litchi fruit development [11]. They started by introducing litchi fruit development and sugar accumulation during aril development, and then explained the current understanding of sugar transport and sugar transporters in litchi fruit [11]. They also reviewed the possible functions of some key enzymes in sugar metabolism in litchi fruit, such as sucrose phosphate synthases, sucrose synthases, and invertases [11]. Lastly, they discussed genes involved in sugar signaling, potentially contributing to fruit abscission [11].

Sugar transport and regulation are complex processes that often vary from species to species. The above studies have made limited progress in understanding molecular mechanisms underlying sugar transport and regulation in those different species, likely due to limitations in the genetic tools that are available, but the results reported in this issue can provide valuable information for future studies.
